# On the Conveyance of Asiatic Cholera from Hindoostan, through Central Asia and Persia, to Russia and England, from 1817 to 1831

**Published:** 1868-05

**Authors:** John C. Peters

**Affiliations:** New York


### ON THE CONVEYANCE OF ASIATIC CHOLERA FROM HINDOOSTAN, THROUGH CENTRAL ASIA AND PERSIA, TO RUSSIA AND ENGLAND, FROM 1817 TO 1831.

---

By JOHN C. PETERS, M. D., of New York.

---

Asiatic, or Hindoostanee cholera, has always originated in India or Hindoostan, and has been conveyed from thence repeatedly to almost all parts of the world. The cholera of 1817 originated at or near the mouth of the Ganges, followed up the course of that river to the northwest, towards its source in the Himalaya Mountains, then pursued the great trunk road of trade and traveled through the cities of Delhi and Lahore, to the Punjaub, or extreme northwestern province of Hindoostan; passed through the Himalaya Mountains along the line of the Cabul river to the city of Cabul; thence followed the course of the caravan trade between Russia and Asia, through the great cities of Central Asia and Independent Tartary, viz.: Balk, Bokhara, and Khiva, to the towns of Astrakhan and Orenburg, in European Russia.

Another great route of cholera was from Bombay, in 1821, to Muscat, at the foot of the Persian Gulf; thence up the Gulf to Bushire; from there through Central Persia to Ispahan and Teheran, and up the Caspian Sea till it reached Astrakhan, in Russia, near the mouth of the Volga, in 1823, when 216 cases, and 144 deaths occurred. The Russian Government established a most rigid quarantine against Astrakhan, and saved the rest of Europe. From Teheran, to Persia, near the foot of the Caspian Sea, it also followed the line of travel up between the Black and Caspian Seas, to Tabrez and Tiflis, in the Caucasus, and reached Astrakhan from this (third) direction. But the precautions of the Russian authorities again prevented this, and from 1823 to 1829, we find European medical writers speaking of cholera as an epidemic which had passed away, and expressing the hope that it would never return.

But in 1827, cholera received a fresh impulse in North-western Hindoostan, and was again carried by caravans from Lahore, in the Punjaub, through the Himalaya Mountains, by way of Attock and Peshawar, to Cabul, Balk, Bokhara, and Khiva, and reached Orenburg, in Russia, on the 29th of August, 1829.

About the same time, it was again carried from Bombay up the Persian Gulf, and again arrived at Teheran, in the northernmost part of Persia, in 1829, whence it was forwarded up the Caspian Sea to Astrakhan, by the 19th of July 1830.

Thus Astrakhan and Orenburg, in Russia, were the first towns in Europe ever attacked by cholera, and if it is asked why this should be, the answer is very easy.

Orenburg is situated on the Ural river, some distance above its mouth, by which it empties into the northern end of the Caspian Sea. The Ural river forms part of the boundary line between Russia and Asia, and Orenburg is built on both sides of this stream, with two separate bazaars—one European, the other Asiatic—each located on the side of the river belonging to their respective countries. The European bazaar has 180 shops; the Asiatic 492. In fact, Orenburg is the chief emporium of the Russian trade with Central Asia, large caravans arriving yearly from Bokhara and Khiva, with jewels, gold, silk, cotton, cashmere shawls, indigo, tea, and other goods, to the value of over £1,500,000. Russia sends back the same amount of iron, cutlery, and other manufactures. More than 3,000 camels are employed in the transport of cast iron articles alone, consisting mainly of pots, kettles, and water cans. Indeed, there is no house, and even no tent, in all Central Asia, where there is not some article of Russian manufacture. The most active trade and correspondence is also kept up between the two countries, for many wealthy Asiatic merchants make constant trips to the Russian Fair of Nishni Novgorod, and frequently travel on to Moscow and St. Petersburg. Orenburg is also the principal Russian military station for operations against Central Asia, for in 1829, the town had only 11,000 inhabitants, of which number 6,000 were soldiers, and in 1839, no less than 20,000 men and 10,000 camels, set out from this city on a campaign against Khiva. Hence we are not surprised to find that cholera first declared itself in the Russian garrison at Orenburg. It prevailed from the 26th of August, 1829, to the 20th of November, and about 1,000 persons had the disease, out of a population of 11,000, but several hundreds only succumbed. It was checked by the cold weather, and by the middle of February, Russia was free of the disease.

Astrakhan is the great rival of Orenburg for the Asiatic trade. Its population is composed of Russians, Armenians, Cossacks, Tartars, Calmucks, Hindoos, and the Asiatic tribes. In fact, it consists of almost all nations of Europe, Asia, and of all creeds. It has mosques for the Mahommedans, temples for the Hindoos, as well as churches for the Christians. It has over 100 large manufacturing establishments, extensive salt works, and flourishing fisheries on the Volga and Caspian Sea. It is likewise one of the principal naval depots, not only for the Caspian and Aral Seas, but also for many lakes and rivers of Central Asia. Numerous sailing vessels and steamers are constructed and armed at Astrakhan for the lakes and rivers of Central Asia.

Cholera was introduced into Orenburg by merchants, travelers, caravans and soldiers, but it came to Astrakhan by sailing vessels, especially from the port of Bakon, on the southwest side of the Caspian Sea. In ten days, 1,290 persons were seized with cholera in Astrakhan, of whom 430 died.

From Astrakhan it extended up the river Volga the distance of 420 miles, in less than a month, and is supposed to have traveled nearly 1,500 miles from Persia, in three and a-half months, in order to reach Nishni Novgorod and Moscow.

It is said that many persons fled from Astrakhan, along the course of the Volga, and carried the disease with them; for in ten different towns along this stream, the first victims were navigators of the Volga, or others arriving from places where the disease already raged. Some distance above its mouth the Volga bends abruptly to the West, and approaches the river Don, which makes an equally marked elbow towards the East, and affords a facile and favorite place of trade for the inhabitants on the two rivers. Don Cossacks visited the Volga for the purpose of trade, contracted the disease, returned to the Don, where some of them died in September, and the cholera commenced to travel down the river Don towards its mouth in the Sea of Azof, and thus spread to the Black Sea, at the same time that it was ascending the Volga to Nishni Novgorod and Moscow. No less than 54,000 Don Cossacks contracted the disease in 1830, and 31,000 of them died. Other Cossacks were drafted in the Russian army, which was going to the Polish war, and with this army, cholera entered the southeast corner of Poland, and was carried northwest to Warsaw, from thence into Prussia. It is very significant that the little Moravian town of Sarepta is situated on the great elbow of the Volga, in the very midst of the Don Cossack district, and has never contracted the cholera. The inhabitants are always very cleanly, and always institute a most rigid quarantine against cholera.

By the 27th of August, the pestilence reached Nishni Novgorod, high up on the Volga. Some say it came from Orenburg, in the East; others from Astrakhan, in the South. It probably arrived almost simultaneously from both places. The chief importance of Nishni Novgorod, is derived from the world-famed fair which is held there annually, and continues during the whole of July and August. It is visited by from 300,000 to 400,000 traders, and the value of the goods exhibited averages $50,000,000, of which more than one-tenth part comes from China, Persia, Central Asia, and India. In fact, Indian, Persian, and Chinese silks, teas, and furs, form some of the principal articles of Russian foreign trade, most of which reach it from Orenburg and Astrakhan. The vessels engaged in taking cargoes in and out are so numerous that the waters of the Oka and Volga rivers, at the confluence of which Nishni Novgorod is situated, are literally crowded and choked with the mass of shipping. The number of Asiatic visitors is also so great that Mahommedan mosques and Hindoo temples are placed in company with the Russian churches within the precincts of the fair.

By the 15th of September, 1830, the cholera reached Moscow, due west of Nishni Novgorod and Orenburg, 50,000 persons fled the city. But up to January, 1831, 9,000 of those who were left were attacked, and more than one-half of them died. Moscow is the seat of the principal Russian manufactories, of which not less than 484 are in active operation. It is also a great centre of internal commerce with Riga, on the Baltic.

From Moscow it was conveyed, early in May, 1831, to Riga, and it is distinctly stated that it was brought to the headwaters of the river Duna, at the mouth of which Riga is situated, and was carried up the stream to that city. Immense alarm arose, and on June 3d, no less than 60 vessels fled in haste from Riga, four of which were destined for England, especially for Sunderland, on the east coast. By October 26th, 1831, an official English report declared that 306 cases and 94 deaths had already occurred in Sunderland, and the first case of cholera in London occurred in the person of a man from Sunderland. From London it was carried to Dover, and over to Calais and Paris, in France. From England it was carried to Ireland, and in the spring of 1832, vessels from Dublin and Cork, conveyed the disease over the Atlantic to Quebec, in the manner stated in the October number of your journal.

From Moscow it was also carried to St. Petersburg, on the 26th of June, 1831, and Drs. Russell and Barry, of the Indian service, who had been familiar with cholera in Hindoostan, were sent by the English Government to observe the disease. A triple cordon of troops were placed around St. Petersburg to keep out the cholera; but Drs. Russell and Barry stated that the first case occurred in a person who came down the river Neva, in a bark with goods from Moscow; the second in an individual who had been on board the bark upon its arrival; and the third in a soldier who had mounted guard on the boat to prevent any intercourse with the shore. From St. Petersburg it was carried down to Cronstadt, on the Baltic, and a new current of the disease was then let loose to visit Hamburg, Bremen, Denmark, Norway, Sweden, and England.

It is well to add that Sunderland, which is only 20 miles from Newcastle, had over 500 vessels engaged in the coal trade, with ports in the Baltic, in 1831; that Riga has the largest commerce of any other port in Russia, except St. Petersburg, and that Drs. Russell and Barry also state that the disease was propagated in St. Petersburg, immediately upon the arrival of several thousand passengers and boatmen, who had come from infected places in the interior of Russia, or had been exposed to infection on the passage on board these vessels.

